# A New Distance Stereotest by Autostereoscopic Display Using an Eye-Tracking Method

**DOI:** 10.3389/fbioe.2022.799744

**Published:** 2022-06-20

**Authors:** Li-Qun Cao, Yuan-Qing Wang, Yuan Gao, Bi-Ye Zhou, Xue-ling Li, Ke-Qiang Shen, Bin Xu, Ming-Gao Li

**Affiliations:** ^1^ Ophthalmology Department, The Sixth Medical Center of Chinese PLA General Hospital, Beijing, China; ^2^ Senior Department of Ophthalmology, The Third Medical Center of Chinese PLA General Hospital, Beijing, China; ^3^ School of Electronic Science and Engineering, Nanjing University, Nanjing, China; ^4^ Naval Medical Center, PLA, Shanghai, China; ^5^ Nautical and Aviation Medicine Center, The Sixth Medical Center of Chinese PLA General Hospital, Beijing, China; ^6^ Beijing Jiacheng Shixin Digital Medical Technology Co., Ltd., Beijing, China

**Keywords:** stereotest, stereoacuity, random-dot stereogram, autostereoscopic display, eye tracking

## Abstract

**Objectives:** This research aimed to present a novel glasses-free distance random-dot stereotest system (GFDRDSS) using an eye-tracking method.

**Methods:** A single-view autostereoscopic display applying a backlight control system combined with an eye-tracking method and the corresponding random-dot stereotest software were developed to create a GFDRDSS with a viewing distance of 5 m. The stereoacuity of 12 subjects with normal eye position was evaluated using the Randot Stereotest, Stereoscopic Test Charts vol. 3 (Yan’s Charts), Distance Randot^®^ Stereotest, and GFDRDSS.

**Results:** The GFDRDSS could provide distinct and stable glasses-free stereoscopic perception even while the subject was moving their head. It could evaluate binocular disparities of 40–2,400 arcsec. Eleven subjects with normal near visual acuity had fine near stereovision (20–60 arcsec) using the Randot stereotest and Yan’s Charts. Under refractive correction, 10 subjects had fine stereovision (≤60 arcsec) using the GFDRDSS at a distance of 5 m, and 9 had fine stereovision using the Distance Randot^®^ Stereotest at 3 m. Other subjects described the 100 arcsec-level stereograms correctly. The results exhibited a concordance of stereoacuity within one degrade between the two distance stereotests.

**Conclusion:** The proposed GFDRDSS can alternately project a couple of random-dot stereograms to the subjects’ eyes and provide a glasses-free distance stereotest, which showed good concordance with the Distance Randot^®^ Stereotest. More data are needed for statistical studies.

## 1 Introduction

Our eyes are separated horizontally, and thus, each eye has a slightly horizontally disparate view in two dimensions of the three-dimensional (3D) world. Stereopsis is the ability to fuse images that stimulate horizontally disparate retinal elements within Panum’s fusional area, resulting in a binocular appreciation of visual objects in depth (Bhola, 2006). As the highest form of binocular coordination, stereopsis is necessary to see the world in three dimensions; it also plays a major role in visuomotor skills (Von Noorden and Campos, 2002), and it involves near (reading distance) and distance stereovision. For more than 30 years, our team has continuously worked toward practical stereotests, following the lead of Shao-ming Yan from the Sixth Medical Center of the Chinese PLA General Hospital (formerly the Navy General Hospital). In 1985, Shao-ming Yan and Zhu-ying Zheng published the first random-dot stereotest (Stereoscopic Test, People’s Medical Publishing House) in China, which could be read at 0.4 m using red–blue glasses. The second edition (Digital Stereoscopic Test Charts, People’s Medical Publishing House) was published in 2006. In 2016, we developed the glasses-free third edition with the lenticular lens sheet (Stereoscopic Test Charts, Vol. 3; People’s Medical Publishing House). Using these experiences in the design of reading distance card-based random-dot stereotests, we attempted to explore a new distance stereotest based on the 3D stereoscopic display technology.

Assessing distance stereoacuity has been advocated as a measure of treatment programs for intermittent exotropia (transitional strabismus between exophoria and constant exotropia) (Holmes et al., 2007). It is also an important assessment for visually guided hand movements (Levi et al., 2015), and it is valuable for pilots, drivers, athletes, and other special occupational groups. For measuring distance stereoacuity in a clinic, the Distance Randot^®^ Stereotest (version 2; Stereo Optical Company, Inc., Chicago, IL, United States) and Frisby–Davis distance (FD2) stereotest are available. The Distance Randot^®^ Stereotest (Wang et al., 2010) is a polaroid vectographic random-dot test designed to examine stereoacuity at a distance of 3 m. It contains eight shapes (two at each disparity level) and can measure stereoacuity of 400–60 arcsec. With only two shapes at each disparity level, it is likely that subjects would be able to remember the results, and the polarized glasses may disturb the brightness and contrast of the stereographs. FD2 uses four real objects presented inside an open-fronted illuminated box, which is presented at a distance of 6 m. The stereoacuity is estimated by identifying which of the four figures appears to protrude. As a real depth test, FD2 is a valuable distance stereotest, but the problem of monocular cues cannot be ignored (Holmes and Fawcett, 2005).

Three-dimensional display-based stereo tests have been reported in recent years. In 2011, Jongshin Kim (Kim et al., 2011) developed a 46-inch polarized stereoscopic monitor with a background luminance of 250 cd/m^2^ and a resolution of 1,920 × 1,080 pixels in order to test contour-based distance (3 m) stereovision. Davide Gadia (Gadia et al., 2014) presented a software-based stereoacuity test at the distance of 1 m using a 21-inch display (1,600 × 1,200 pixels) with circular polarized filters. Angelo Gargantini (Gargantini et al., 2014) measured random-dot stereoacuity using a 3D monitor (refresh rate = 120 Hz) using NVIDIA active liquid-crystal display (LCD) shutter glasses from a distance of 0.4–2 m. Sang Beom Han (Han et al., 2015), and subsequently Huang Wu (Wu et al., 2016), developed a shutter glass-type 3D stereoscopic display to evaluate stereoacuity (testing distance of 0.5 and 3 m by Sang Beom Han and 4.1 m by Huang Wu). Computer-based stereotests have overcome some of the deficiencies of traditional card-based tests. Their variable stereograms and stereoacuity-level settings avoid problems with subjects remembering the results in repeat examinations, which is known as the learning effect (Frisby and Clatworthy, 1975). The self-luminous stereograms are not as affected by environmental luminance as the card-based tests (Livingstone and Hubel, 1994). However, the interference of the polarized or 3D shutter glasses remains a problem. Based on our past experiences, polarized glasses reduce the brightness and definition of the test cards, especially in dark environments. Monitors with shutter glasses may be influenced by ambient light. In addition, sharing the same pair of 3D glasses between multiple subjects creates the potential for cross-infection.

In 2015, Jonghyun Kim presented a glasses-free random-dot stereotest (Kim et al., 2015) using a multi-view display system (observation distance = 1.38 m) by placing a four-view parallax barrier system in front of a display panel to show random-dot multigrams that were generated. This eliminated the negative effects of 3D glasses on stereoacuity evaluation. However, stereoscopic perception is affected by the subject’s observation position, as with other autostereoscopic displays based on the parallax barrier or lenticular lens sheet system. If the subject cannot view the display panel from the ideal position, the stereoscopic perception will not be distinct, and monocular motion parallax as a result of the movement of the eyes may also disturb the examination.

In this study, a new autostereoscopic display with a backlight control system and eye-tracking system is introduced. In addition, we developed a corresponding random-dot stereotest (RDS) software. The two parts mentioned above constitute the glasses-free distance random-dot stereotest system (GFDRDSS). In contrast to the traditional autostereoscopic displays that subjects need to observe passively from an ideal viewing position, GFDRDSS produces bidirectional random-dot stereograms sequentially, which can actively follow the subjects’ eyes. In this study, we implement the system and present clinical data from 12 subjects. From these results, it may be possible to uncover whether the novel system can provide a distance glasses-free stereotest that can be used in the clinic and decide whether it is necessary to conduct statistical studies.

## 2 Methods

### 2.1 GFDRDSS Configuration and Design

#### 2.1.1 Autostereoscopic display system

The development environment of autostereoscopic display includes an Intel Core i7 4720HQ CPU and NVIDIA GTX970 graphics card, which are controlled by Microsoft Windows 7.0.

**FIGURE 1:**
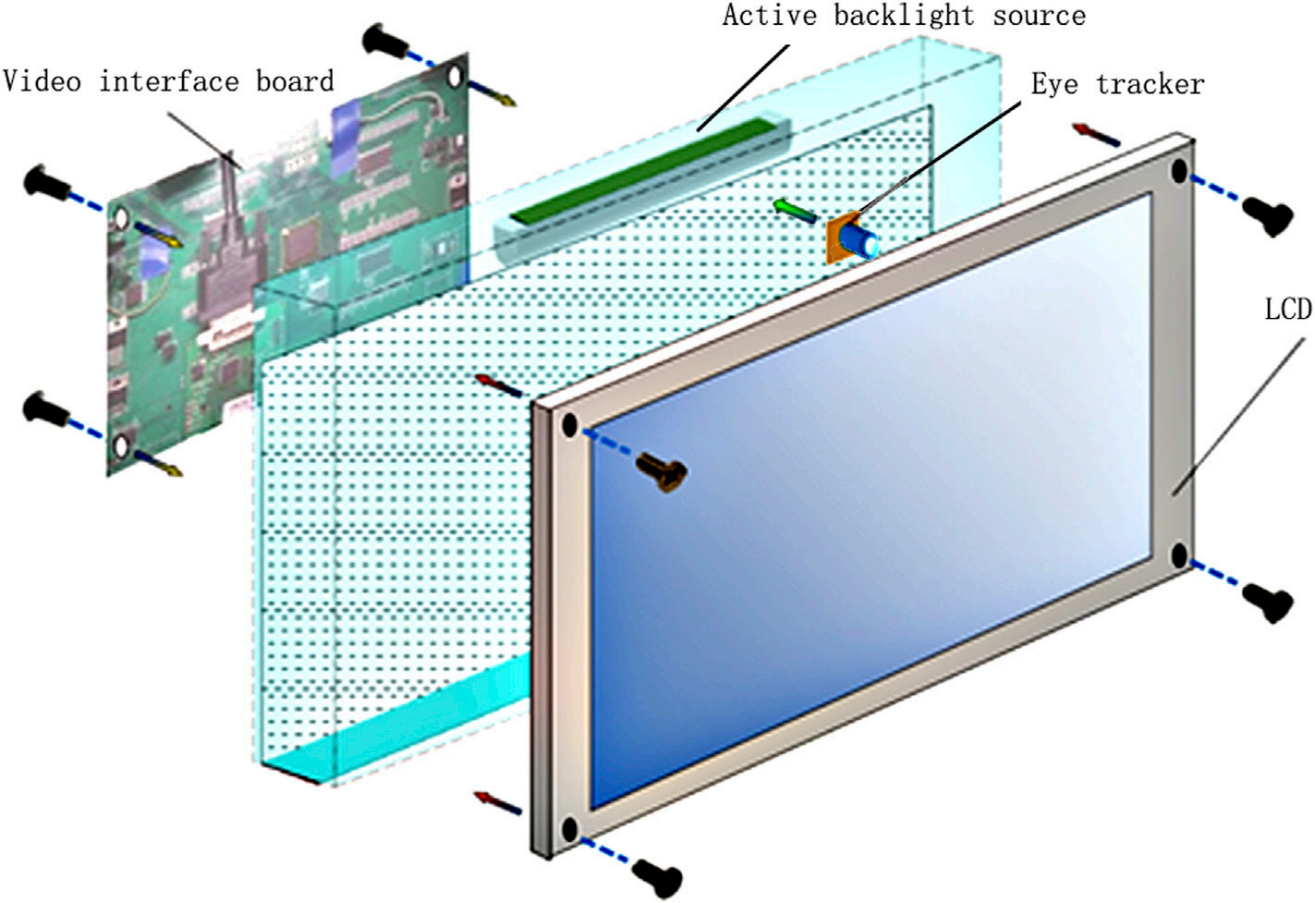
A schematic illustration of the autostereoscopic display system with the eye tracker in the GFDRDSS.

The overall structure of the system consists of four major parts (Figure 1): a video interface board, active backlight source, eye tracker, and an LCD (display size: 23 in, resolution: 3,840 × 2,160 pixels, and frame rate: 120 Hz). Channel separation of the parallax images, meaning that the left eye and right eye each get the corresponding image, is realized by the cooperation of the four parts, which utilize the temporospatial multiplexed technique. In the time domain, the LCD displays a pair of left and right parallax images frame by frame. In the space domain, the active backlight source projects the displayed image to the positions of the viewer’s eyes through special optical components (Xue et al., 2014). In order to form the correct stereo effect, the projection of the backlight needs to be focused onto the viewer’s eye position. Hence, the eye tracker is used, which obtains the viewers’ left and right eye positions in real time and sends them to the active backlight source (Jin et al., 2015). In addition, the active backlight source must be synchronized with the LCD. Therefore, a synchronization signal is extracted from the video interface board, and it is offered to the backlight and the LCD. In this way, full resolution autostereoscopic images can be achieved (Xu et al., 2019a).

**FIGURE 2:**
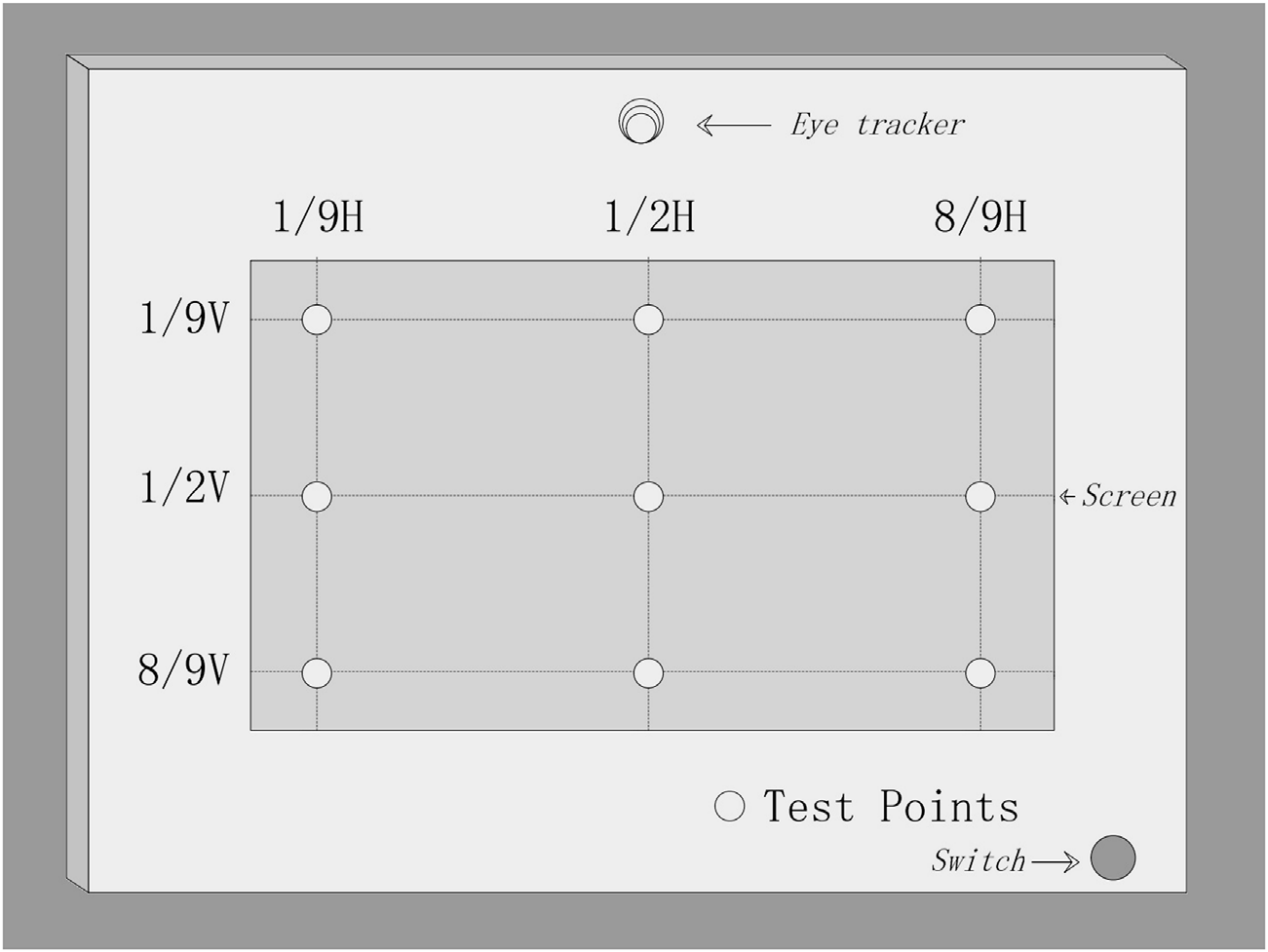
Distribution diagram of the nine observation points on the screen selected to measure the brightness of the autostereoscopic display.

The brightness of nine points on the screen (Figure 2) was measured using a TES-1336A digital luminance meter vertically when the whole screen displayed a 3,840 × 2,160 full-white pixel signal. During each test, the accuracy and stability of the eye-tracking system in locating subjects’ faces and eyes were recorded by the presenter. When a cluttered background disturbed the eye location program, the presenter drew a straight line from the top left to the bottom right of the subject’s face in the eye-tracking window using a mouse, and then the face and eyes were tracked promptly.

#### 2.1.2 Random-Dot Stereotest Software

**FIGURE 3:**
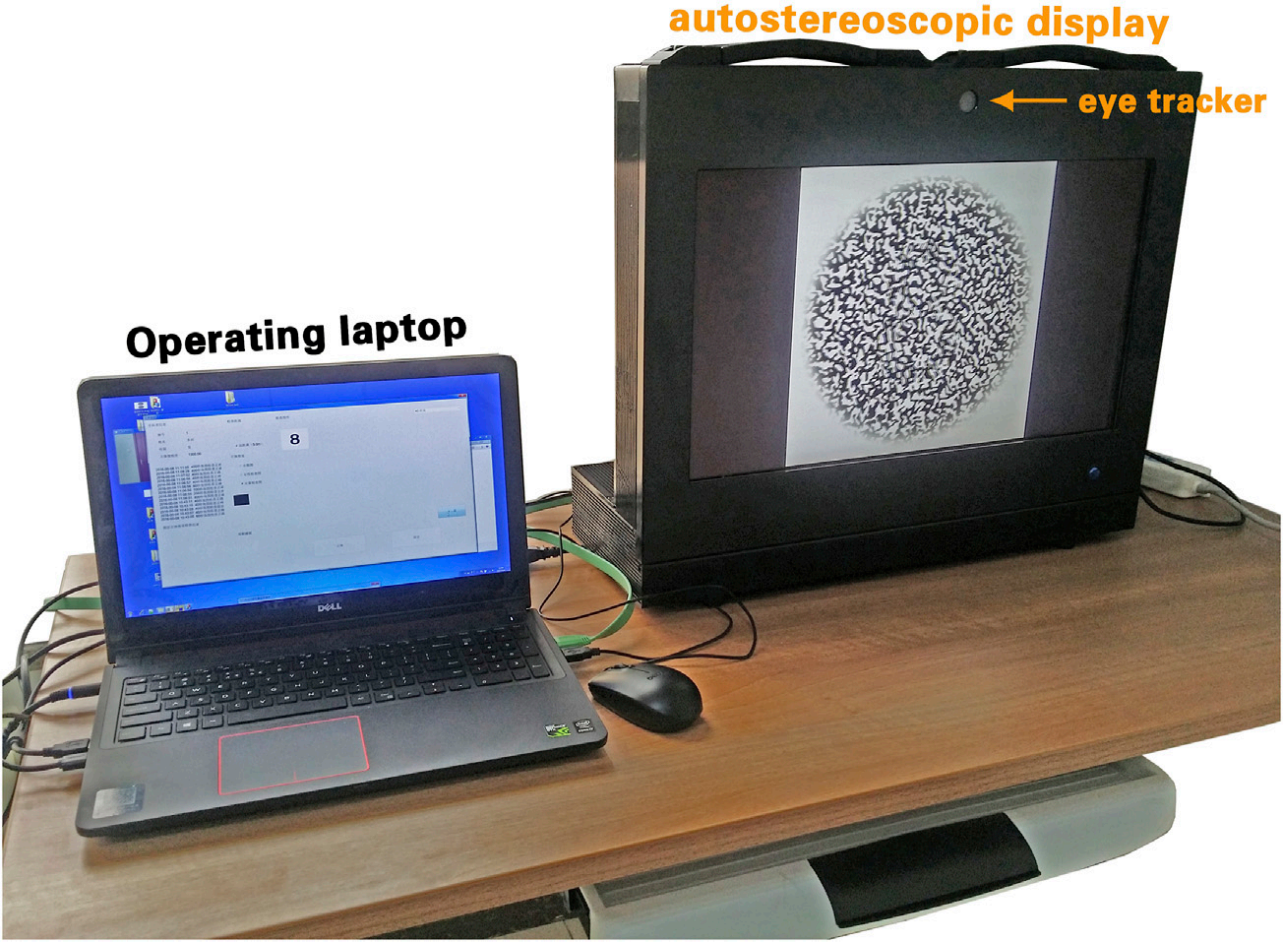
A view of the GFDRDSS. It consists of a laptop computer for the operating system and an autostereoscopic display with an eye-tracker placed on top of it.

A notebook computer system was designed using a laptop (ASUS G750Y47JX, 17.3 inches 16:9 full HD 3D [1,920 × 1,080, 120 Hz]) running Microsoft Windows 7.0. The RDS software was implemented using the C++ compiler and libraries combined with the Lua language under Microsoft Windows 7.0. The system was operated on a single computer, and a data transmission network environment was supported. The software was developed using Microsoft Visual C 7.0 on a Directx SDK platform. Structured query language was used for the design of the database query invocation, and MySQL Cluster was employed as the database service. The stereographs were drawn using Adobe Photoshop CS5. The random-dot stereogram design was based on Yan’s Stereoscopic Test Charts. The implemented 3D display was used for the stereographic demonstration to the subjects, while an additional 14-inch laptop was used for system management by the presenter (Figure 3).

Three items were set for the test: 1) the teaching graph included an 800 arcsec random-dot circle and a similar contour circle beside it. The diameters of both circles were 15 cm. Before the formal stereotest, it was suggested that subjects first experience the stereoscopic perception using the teaching graph. 2) The screening test established the presence of gross stereopsis to screen for stereoblindness. In this graph, there were three random-dot stereograms, that is, a large circle, a medium square, and a small pentagon, with a single center, which looked a bit like a triple-layer birthday cake. The binocular disparity of the stereograms was 800, 1,600, and 2,400 arcsec, respectively. A subject who cannot recognize any of these stereograms is suspected to have stereoblindness and should be referred to an ophthalmologist for further examination. 3) The stereoacuity test included random-dot stereograms that were generated with six disparity levels (800, 400, 200, 100, 60, and 40 arcsec). Ten different stereographs were designed based on simple geometric shapes, for example, square, circle, triangle, rectangle, and cross; capital letters; and Arabic numbers for each level.

**FIGURE 4:**
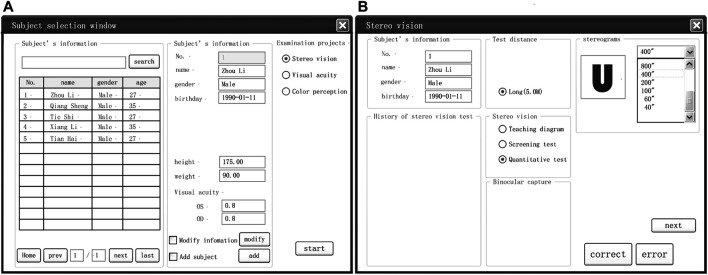
An example of interfaces designed for the GFDRDSS. **(A)** General information registration and inspection item selection interface. **(B)** Stereovision examination interface.

For the convenience of clinical application, a management program was developed for the presenter interface (Figure 4). The presenter begins by entering the subject’s information, that is, name, age, examination date, and visual acuity. They can then select the teaching graph, screening test, or quantitative test by pressing the corresponding button. Each subject was initially shown the teaching graph to allow them to experience the stereoscopic perception. In the quantitative test, six levels of disparity could be tested, and it was recommended that the presenter begin the quantitative test with the 800 arcsec level. If the subject correctly names any shapes at this level (up to five different diagrams are randomly provided for each level), the subject then moves on to the 400 arcsec level. The test proceeds in this manner until the subject is unable to describe any of the five figures in a level. If a subject had stereoscopic perception but could not accurately describe the stereoscopic shapes, the presenter could give hints about the range of the shapes, for example, capital letters, Arabic numerals, or simple geometric shapes. The lowest level that a subject could recognize was recorded as their stereoacuity.

### 2.2 Evaluation Procedures

#### 2.2.1 Subjects

The clinical examination was conducted at the Sixth Medical Center of the Chinese PLA General Hospital in China. A total of 12 volunteers (seven males and five females aged 20–52 years) who have never experienced stereotests were included. Measurements of distance visual acuity were obtained using a standard logarithmic visual acuity chart at 5 m and a Jaeger chart at 40 cm, which were both recorded as logα, where α represents the visual angle, using arcmin as the unit. The inclusion criteria were as follows: 1. corrected visual acuity (with their own daily refractive glasses) of ≤0.3 in each eye, as tested by the standard logarithmic visual acuity chart 2. no manifest tropia at distance or near fixation on the Hirschberg test, alternate cover test, cover–uncover test, or simultaneous prism and cover test. The exclusion criteria were as follows: 1. any ocular or systemic disease or 2. any history of ocular or neurological disorders. Their gender, age, distance visual acuity (with and without their glasses), refractive condition, and near visual acuity are listed in Table 1.

**TABLE 1:** The general conditions and visual acuity (VA) of 12 subjects.

Subjects	Gender	Age	VA (5 m)				Refractive condition (DS^c^)		VA (0.4 m)	
			Not corrected		Corrected					
			OD^a^	OS^b^	OD	OS	OD	OS	OD	OS
1	Male	20	0.05	0.05					0	0
2	Female	21	0.05	0.05					0	0
3	Male	23	−0.1	0					0	0
4	Female	23	0.15	0.1					0	0
5	Male	23	0.15	0.05					0	0
6	Male	38	0	0					0	0
7	Male	47	0.1	0	0	0	−0.50		0	0.1
8	Female	27	1	0.70	−0.1	−0.1	−3.50	−3.50	0	0
9	Male	32	1	1	0	−0.1	−3.00	−3.50	0	0
10	Female	45	1	1	0	0.1	−2.75	−4.25	0	0
11	Female	46	−0.1	1	−0.1	0.1		−3.00	0	0
12	Male	52	1	0.1	0.1	0.1	−2.75		0.5	0.4

a OD (oculus dexter): right eye.

b OS (oculus sinister): left eye.

c DS: diopter of spherical equivalent.

#### 2.2.2 Stereotests

All the subjects first completed near stereotests and then distance tests. The test was performed for each subject according to their subject number with an interval of ≥5 min between two tests for each subject. The subjects with ametropia were instructed to wear their corrective glasses during every stereotest.

##### 2.2.2.1 The Glasses-Free Distance Random-Dot Stereotest System Test

A member of the research group with excellent stereovision was asked to experience the test first to confirm that the system was running correctly. The subject sat at a distance of 5 m from the autostereoscopic display with their eyes in the horizontal position and focused on the center of the display. A presenter started the test procedure, activated the eye-tracking program, and ensured that both of the subject’s eyes were detected and tracked. The stereotest then began with the teaching graph and progressed to performing the stereoacuity test, starting at the 800 arcsec level, until the subject’s stereoacuity was recorded. During eye tracking and stereotesting, any problems and the required treatment methods were recorded by the presenter.

After completing the GFDRDSS test, the subjects were asked to review the teaching stereogram. The difference in its stereo effect was then compared between the monocular and binocular view and with the eye-tracking system on and off. After the process was completed, the subjects were invited to answer five questions about any discomfort they experienced and the stability of the stereoscopic figures they perceived (Table 2).

**TABLE 2:** Results of questionnaires from subjects after GFDRDSS examination.

Questions and answers	Frequency (%)
1. Have you ever experienced any discomfort during the test?	
a. Nothing	11 (91.67)
b. Unbearable glare	0 (0)
c. Mild glare that does not affect the examination	1 (8.33)
d. Unbearable eye fatigue	0 (0)
e. Mild eye fatigue that does not affect the examination	1 (8.33)
f. Dizziness, palpitations, or nausea	0 (0)
g. Any other discomforts: (please give a brief description)	0 (0)
2. Were the binocular stereoscopic figures you perceived clear and stable?	
a. Clear and stable	12 (100)
b. Clear but flickering	0
c. Too blurred and difficult to identify	0
3. Were the stereoscopic figures still clearly and steadily perceived when you swing your head?	
a. Clear and stable	10 (83.33)
b. Basically stable but can’t be perceived at some moment while swinging head fast	2 (16.67)
C. Unstable. The stereoscopic figures disappeared when head position changes	0 (0)
D. The stereoscopic figures could only be perceived at certain viewing point	0 (0)
4. Can you perceive the stereo circle in teaching graph with a single eye?	
a. Yes	0 (0)
b. No	12 (100)
c. A 2-dimentional circle could be perceived	0 (0)
5. While turning off the eye-tracking system, was there any differences in the stereo circle you perceived?	
a. Clear and stable stereo circle could be still perceived	0 (0)
b. Nothing in 3-dimmention could be perceived	0 (0)
c. The stereo circle could only be perceived at certain viewing points	12 (100)

##### 2.2.2.2 Distance Randot^®^ Stereotest

The subjects wore polarized glasses and viewed the test cards at 3 m from the shapes at the 400 arcsec level. The smallest disparity at which the subject could identify either of the two shapes was recorded as their stereoacuity.

##### 2.2.2.3 Near-Distance Stereotests

Both tests were standardized for 40 cm, and minor variations in distance were permissible. The subjects were tested using circles in the Randot^®^ Stereotest (Stereo Optical Company, 2015) while wearing polarized glasses and without 3D glasses using Yan’s Stereoscopic Test Charts (Yan’s Charts in Table 3), which is a glasses-free random-dot stereotest applying grating stereo printing technology, with eight disparity levels (800–40 arcsec) and a single stereogram for each level (vol. 3, People’s Medical Publishing House).

**TABLE 3:** The stereoacuity of 12 subjects examined by four stereotests.

Subjects	Visual acuity		Randot	Yan’s chart	Distance Randot	GFDRDSS
	OD^a^	OS^b^	Stereoacuity (arcsec/group)	Stereoacuity (arcsec/group)	Stereoacuity (arcsec/group)	Stereoacuity (arcsec/group)
1	0.05	0.05	20/f	40/f	100/m	60/f
2	0.05	0.05	20/f	40/f	60/f	60/f
3	−0.1	0	20/f	40/f	60/f	60/f
4	0.15	0.1	25/f	40/f	100/m	100/m
5	0.15	0.05	50/f	40/f	60/f	60/f
6	0	0	20/f	40/f	100/m	100/m
7	0.1	0	20/f	40/f	400/c	200/m
	0	0	20/f	40/f	60/f	60/f
8	−0.1	−0.1	20/f	40/f	60/f	40/f
9	0	−0.1	25/f	40/f	60/f	40/f
10	0	0.1	20/f	40/f	60/f	40/f
11	0.1	−0.1	30/f	40/f	60/f	60/f
12	0.1	0.1	200/m	200/m	60/f	40/f

a OD (oculus dexter): right eye.

b OS (oculus sinister): left eye.

The stereoacuity results were grouped as one of three levels: fine (f): 20–60 arcsec, moderate (m): 100–200 arcsec, and coarse (c): ≥400 arcsec.

##### 2.3.3 Test Environment

All the stereotests were performed indoors. The room illumination was recorded as 250–400 lux, as measured using a screen luminance meter (SM208 Sanpometer, Shenzhen Sanpo Instrument Co., Ltd., China) at the eye level of each subject.

##### 2.2.4 Evaluation of Results

All data were processed using SPSS 22.0 (IBM SPSS Inc.). The Wilcoxon matched-pair signed-rank test was used to compare the differences between pair of groups. For comparison among different tests, the near and distance stereoacuity tests were grouped into three levels: fine (20–60 arcsec), moderate (100–200 arcsec), and coarse (≥400 arcsec).

## 3 Results

### 3.1 Autostereoscopic Display Evaluation

**FIGURE 5:**
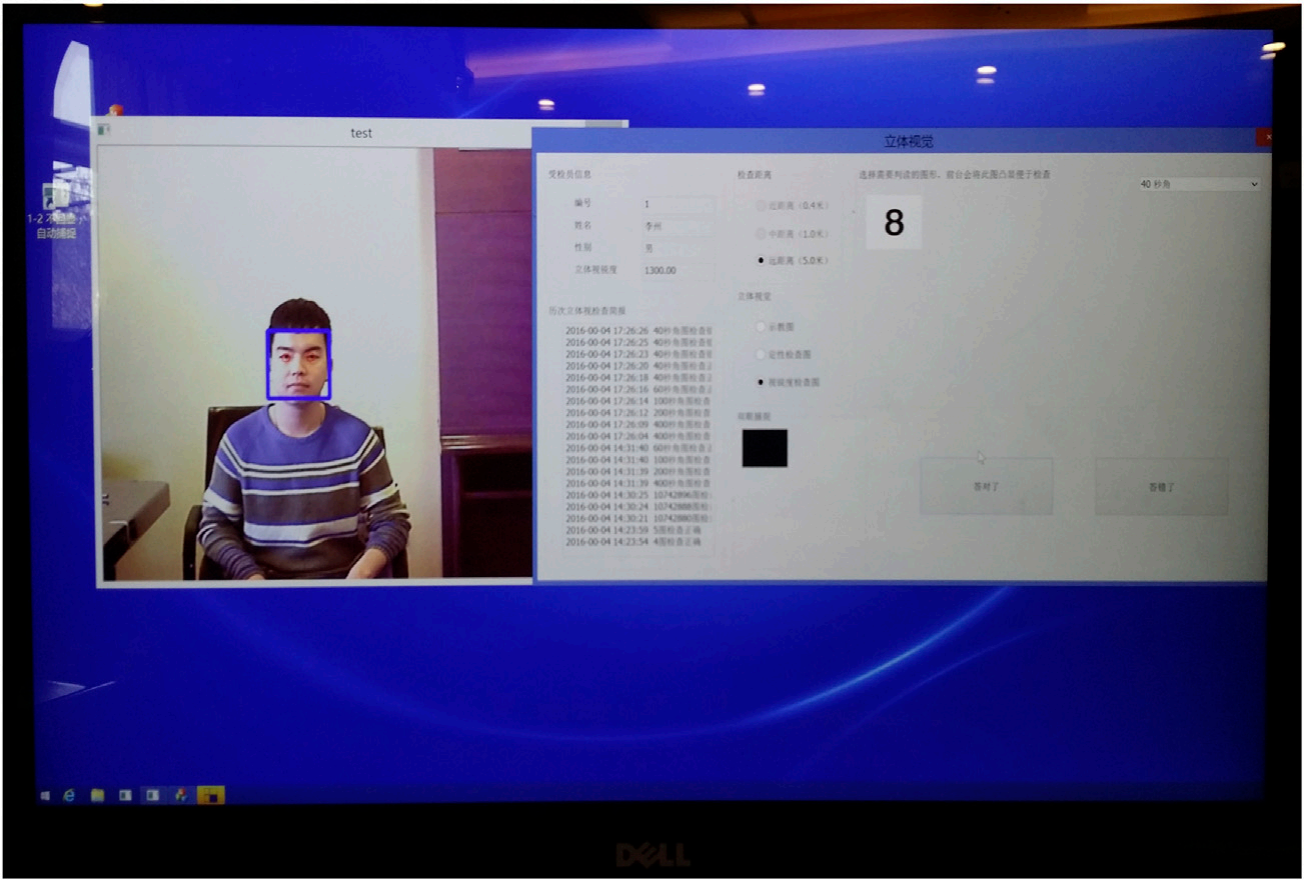
The subject’s face (blue square) and both eyes (red circles) were located by the eye tracker.

The device showed a distinct and stable 3D viewing effect and was switchable between the 2D and 3D modes. The average brightness of the display was 304 cd/m^2^ in the 2D mode and 301 cd/m^2^ in the 3D mode. The eye-tracking system could promptly locate the subject’s face and eyes, and then steadily track both eyes (Figure 5).

### 3.2 The Glasses-Free Distance Random-Dot Stereotest System Examination

The distance stereoacuity of the 12 subjects was tested using the GFDRDSS. The eye-tracking program rapidly located the position of the subject’s face and eyes and stably tracked their eyes, with or without their corrective glasses. The results of the questions answered by the subjects after examination are summarized in Table 2. None of these subjects felt any serious discomfort during the examination. Only one subject (no. 11 in Table 1) complained of mild glare that did not affect her examination. The same subject experienced mild visual fatigue while recognizing stereograms at disparity levels ≤100 arcsec. At 5 m in front of the display, the subjects could not perceive the stereo shape with a single eye, while they could perceive binocular distinct 3D graphics steadily even with natural head movements in the camera view. Only two subjects (nos. 1 and 3 in Table 1) experienced transient disappearance of the stereoscopic figures while their heads were moving rapidly. After the eye-tracking program was turned off, subjects could only clearly distinguish the teaching stereograph at certain viewing points.

The Distance Randot^®^ Stereotest was also applied to evaluate the distance stereoacuity to allow for comparisons between the testing methods. In addition, the subjects’ near stereoacuity was tested using circles in the Randot^®^ Stereotest (2015) and Yan’s stereoscopic test charts (vol. 3), and subject nos. 8–12 were tested while wearing their glasses. The results are summarized in Table 3. Subject no. 7 could only recognize 200–400 arcsec stereograms in both distance stereotests. The subject’s distant visual acuity (logα) of the right eye was corrected from 0.1 to 0 by −0.5 diopter of the spherical equivalent after subjective manifest refraction. After refractive correction, his distance stereoacuity in both the GFDRDSS and Distance Randot^®^ Stereotest was improved to 60 arcsec.

A total of 13 near stereotests were performed on the 12 subjects, and 11 out of 12 subjects exhibited fine near distance stereovision, with the exception of subject no. 12 whose stereoacuity was 200 arcsec (Table 4), and the results of the two near stereotests for this subject were identical. For the distance stereovision evaluation, 10 subjects exhibited fine distance stereovision at 60 or 40 arcsec, and the other two subjects scored 100 arcsec using the GFDRDSS. In the control group, nine subjects passed the Distance Randot^®^ Stereotest at 60 arcsec, and the other three subjects were able to pass at the 100 arcsec level. Of the 13 tests completed by the 12 subjects, 11 out of 13 had concordant scores on the GFDRDSS and the Distance Randot^®^ Stereotest. According to the Wilcoxon signed-rank test, there was no statistically significant difference (*p* = 0.157) between the two distance stereotests, and only two subjects (nos. 1 and 7 without refractive correction) revealed a one grade difference between the two distance stereovision tests (Table 5).

**TABLE 4:** The results of 13 stereotest from 12 subjects examined by Randot and Yan’s Charts (0.4 m).

Randot	Yan’s chart		
	Fine	Moderate	Coarse
Fine	12	0	0
Moderate	0	1	0
Coarse	0	0	0

Fine: 20–60 arcsec, moderate: 100–200 arcsec, coarse: ≥400 arcsec.

*Z* = 0.000, *p* = 1.000, by the Wilcoxon matched-pairs signed-rank test.

**TABLE 5:** The results of 13 stereotest from 12 subjects examined by Distance Randot (3 m) and GFDRDSS (5 m).

Distance Randot	GFDRDSS		
	Fine	Moderate	Coarse
Fine	9	0	0
Moderate	1	2	0
Coarse	0	1	0

Fine: 40–60 arcsec, moderate: 100–200 arcsec, coarse: ≥400 arcsec.

*Z* = −1.414, *p* = 0.157, by the Wilcoxon matched-pairs signed-rank test.

## 4 Discussion

This study provides a novel 3D glasses-free system for distance random-dot stereoacuity measurement. When compared with card-based stereotests, 3D display-based stereotests could provide more variable stereograms and more quantitative methods. Displays equipped with auxiliary 3D glasses (e.g., active shutter glasses or polarized glasses) were first applied to evaluate stereopsis, considering their relatively low crosstalk rate. Crosstalk refers to the incomplete isolation of the left and right image channels so that the content from one channel is partly present in the other (Woods, 2012). A polarized stereoscopic monitor developed by Jongshin Kim realized a contour-based stereotest with a disparity range of 5,000–20 arcsec at a view distance of 3 m (Kim et al., 2011) and brought a promising prospect to this field. However, the inconvenience of wearing glasses and the brightness loss limited their development. Three-dimensional glasses lead to differences between binocular vision in the examination and in daily life, and create a potential risk of cross-infection. Glasses-free 3D displays give us the opportunity to experience stereotests in a more natural and free state. Jonghyun Kim (Kim et al., 2015) made an encouraging attempt at designing such a system by designing a parallax barrier system for a glasses-free random-dot stereotest, which had four or two viewpoints with an interval of 31.25 mm (half of the interocular distance) and an observation distance of 1.38 m. The low crosstalk (6.42% for four viewpoints and 4.17% for two) at the ideal viewing position ensured an ample stereo effect. However, the viewer’s eyes need to be fixed at a certain point. As soon as the eyes leave that point, a significant increase in crosstalk results in impaired stereoscopic perception. To illustrate this point, when we estimated stereoacuity using Yan’s glasses-free stereoscopic test charts, which apply the multi-view parallax barrier (another spatial multiplexed solution besides the parallax barrier), the most important thing is to find the ideal viewing site for the subject, which is easy for normal subjects but not for patients with impaired stereovision or with static tremor. This is a key point that our research has targeted. The perceived brightness loss from polarized glasses and the parallax barrier are also concerns. We intend to provide a 3D display with higher resolution and brightness to improve detection sensitivity.

The proposed autostereoscopic display applying a backlight control system combined with an eye tracking method alternately projects a pair of left and right random-dot images that actively and continuously follow the corresponding eye position of the viewer. It keeps the crosstalk at approximately 6% in the continuous viewing zones of about ±26° (Xu et al., 2019b) (calculation equation in Supplementary Appendix SA). Its crosstalk is consistent with Jonghyun Kim’s parallax barrier system (Kim et al., 2015), while its viewing angle with acceptable crosstalk is significantly expanded compared to traditional autostereoscopic display systems (comparison data of viewing angles from different systems in Supplementary Appendix SB). In addition, it maintains the original physical resolution and brightness by using a spatial-sequential-multiplexed resolution (Xu et al., 2019b). Its definition (3,840 × 2,160) and brightness (301 cd/m^2^) are higher than Jongshin Kim’s polarized stereoscopic display (Kim et al., 2011). These parameters are sufficient for both distance stereotests and visual acuity tests.

In this initial clinical test, the subjects could experience distinct depth perception at any site in the inspection position while moving their head. When the eye tracker was turned off, all the subjects could only perceive the stereo shape at certain viewing points. This demonstrates the importance of the eye tracking method to ensure that stereograms dynamically follow subjects. Feedback from two of the subjects showed that a rapid head swing interferes with the stability of stereoscopic images. Both reported that they deliberately swung their heads quickly to see if the stereoscopic effect would be affected. The backlight tracking speed of the GFDRDSS is set at ≤5 km/h (a normal walking speed). Improving this index could further reduce the interference of subjects’ activities. However, given that most subjects do not violently swing their heads during the exam, the GFDRDSS should be adequate for a clinical stereotest. Recognizing a random-dot stereogram requires a set of higher cognitive processes, not just correct stereopsis. Therefore, it could hardly be disentangled by any observers on the first view. However, it has the remarkable advantage of hiding the outline of the feature in monocular views (Westheimer, 2013). In order to eliminate the monocular clues from contour-based stereograms, we developed RDS software displayed by our glasses-free 3D monitor to present a GFDRDSS for practical distance stereoacuity measurement. The results show that none of the subjects could perceive the stereo shape with a single eye. We designed a teaching graph that would help those subjects who have normal stereovision but have trouble recognizing random-dot stereograms. The Distance Randot^®^ Stereotest and most other distance computer-based stereotests (Kim et al., 2011; Han et al., 2015; Wu et al., 2016) have a viewing distance of 3–4 m, but we set the examination distance to 5 m, referring to the corresponding eye position and visual acuity tests.

Our results show that the GFDRDSS can provide a distance stereoacuity evaluation at 5 m free from perceivable monocular clues and 3D glasses. Most of the subjects completed the entire test in 1 min. It provides a distinct and stable stereopsis perception at any site in the camera view of the eye tracking system. The clinical examination showed that 11 out of the 12 subjects had fine near distance stereovision, with the exception of subject no. 12 (near stereoacuity of 200 arcsec) who had poor near visual acuity. As to the distance stereovision, all 12 subjects could recognize stereograms at 100 arcsec under refractive correction. A total of 10 subjects exhibited fine stereovision using the GFDRDSS and nine exhibited fine stereovision using the Distance Randot^®^ Stereotest. These results show that the near and distance stereovision of these subjects with normal eye position and visual acuity at corresponding distances were basically identical and normal. They also reveal the concordance between the proposed GFDRDSS and the Distance Randot^®^ Stereotest. The consistency of these test results reached 84.6% over the 13 tests completed by the 12 subjects (11 out of 13). Both of the subjects with discordant scores (nos. 1 and 7 without refractive correction) had scores that were one level poorer when using the Distance Randot^®^ Stereotests than the GFDRDSS. One confusing result came from subject no. 7 whose distance stereoacuity was obviously improved with only −0.50 diopter refractive correction in his right eye. A possible explanation may be the weakening senile accommodation of this 47-year-old subject. Previous research has revealed that changes in depth perception of random-dot stereograms with fixed distance (0.3–1.3 m) were independent of the accommodation (Gonzalez et al., 1998). However, the effect of the accommodation on long distance stereoacuity needs further research.

Our study had several limitations. First, the small number of subjects may lead to statistical bias. More statistical data on normal populations and populations with strabismus are currently being collected. Second, the questionnaire design was too simple and lacked a quantitative evaluation index. In order to facilitate comparison with other existing stereotests, our disparity-level settings refer to them. Computer systems have afforded us much more latitude in the setup in the future, including color stereoscopic vision, stereoscopic inspection of binocular asymmetric information input, and binocular perceptual training. There is still plenty to explore.

## 5 Conclusion

We presented a new glasses-free random-dot stereotest named GFDRDSS using an eye-tracking method to evaluate stereoacuity at a distance of 5 m. It eliminates the limitations on the viewing point and the brightness loss of previously reported glasses-free 3D displays. A clinical test with 12 subjects revealed that GFDRDSS showed good concordance with the Distance Randot^®^ Stereotest. The results encourage us to conduct further research. More quantitative data will be brought into future clinical examinations to analyze the validity and reliability of the GFDRDSS.

## Data Availability

The original contributions presented in the study are included in the article/Supplementary Material, further inquiries can be directed to the corresponding author.

## Appendix A: The calculation equation for crosstalk of GFDRDSS.

$$CT_{i}=\frac{\sum_{j\neq i}L_{j}}{L_{i}}$$

By placing a photodetector on the viewing plane in replacement of the eye, the light distribution of the exit pupil, which is the area that the active backlight module projects the displayed image at, can be measured. Crosstalk can be calculated by the present equation.

In the equation, ***L***_***i***_ is the light distribution of the considered exit pupil at a certain position, and ***L***_***j***_ is the distribution of the rest exit pupils at the position. The viewing area is supposed to be where the crosstalk is smaller than a acceptable value that guarantees stereo vision. Therefore, the viewing angle of the autostereoscopic display used in GFDRDSS is ±26°, where the crosstalk is always smaller than 6%.

## Appendix B: A table with comparison data of viewing angles from different autostereoscopic display systems.

According to other literature on autostereoscopic display systems, the viewing angles of some different approach are summarized in above table.

**Table:**

Autostereoscopic display systems	Viewing angle
System in GFDRDSS	±26°
System with freeform surface backlight (FFSB) (Fan et al., 2015)	±23°
System with parallax barrier (Fan et al., 2015)	±12°
System with micro-projection dynamic backlight (Zhao et al., 2021)	±12°
